# Supplementary material for: Complete male-to-female sex reversal in XY mice lacking the miR-17~92 cluster
Source: Nat Commun. 2024 May 7;15:3809. doi: 10.1038/s41467-024-47658-x (PMC11076593; doi:10.1038/s41467-024-47658-x)
Supplement: Supplementary file 1 — Supplementary Information [file 41467_2024_47658_MOESM1_ESM.pdf]

SUPPLEMENTARY MATERIAL

Complete male-to-female sex reversal in XY mice lacking the *miR-17~92* cluster

by

Alicia Hurtado[1,2,3,*], Irene Mota-Gómez[2,*], Miguel Lao[1,*], Francisca M. Real[4], Johanna Jedamzick[2], Miguel Burgos[1], Darío G. Lupiáñez[2,3,#,*], Rafael Jiménez[1,#,*], Francisco J. Barrionuevo[1,#,*].

[1] Department of Genetics and Institute of Biotechnology, Labs. 127 and A105, Centre for Biomedical Research, University of Granada, Armilla, Granada, Spain.

[2] Epigenetics and Sex Development Group, Berlin Institute for Medical Systems Biology, Max-Delbrück Center for Molecular Medicine, Berlin, Germany.

[3] Current address: Centro Andaluz de Biología del Desarrollo (CABD), CSIC/UPO/JA, Seville, Spain.

[4] Research Group Development & Disease, Max Planck Institute for Molecular Genetics, Berlin, Germany.

* These authors contributed equally to this work.

[#] Corresponding authors: Darío G. Lupiáñez (dario.lupianez@csic.es); Rafael Jiménez (rjimenez@ugr.es); Francisco J. Barrionuevo (fjbarrio@go.ugr.es).

Supplementary Figures

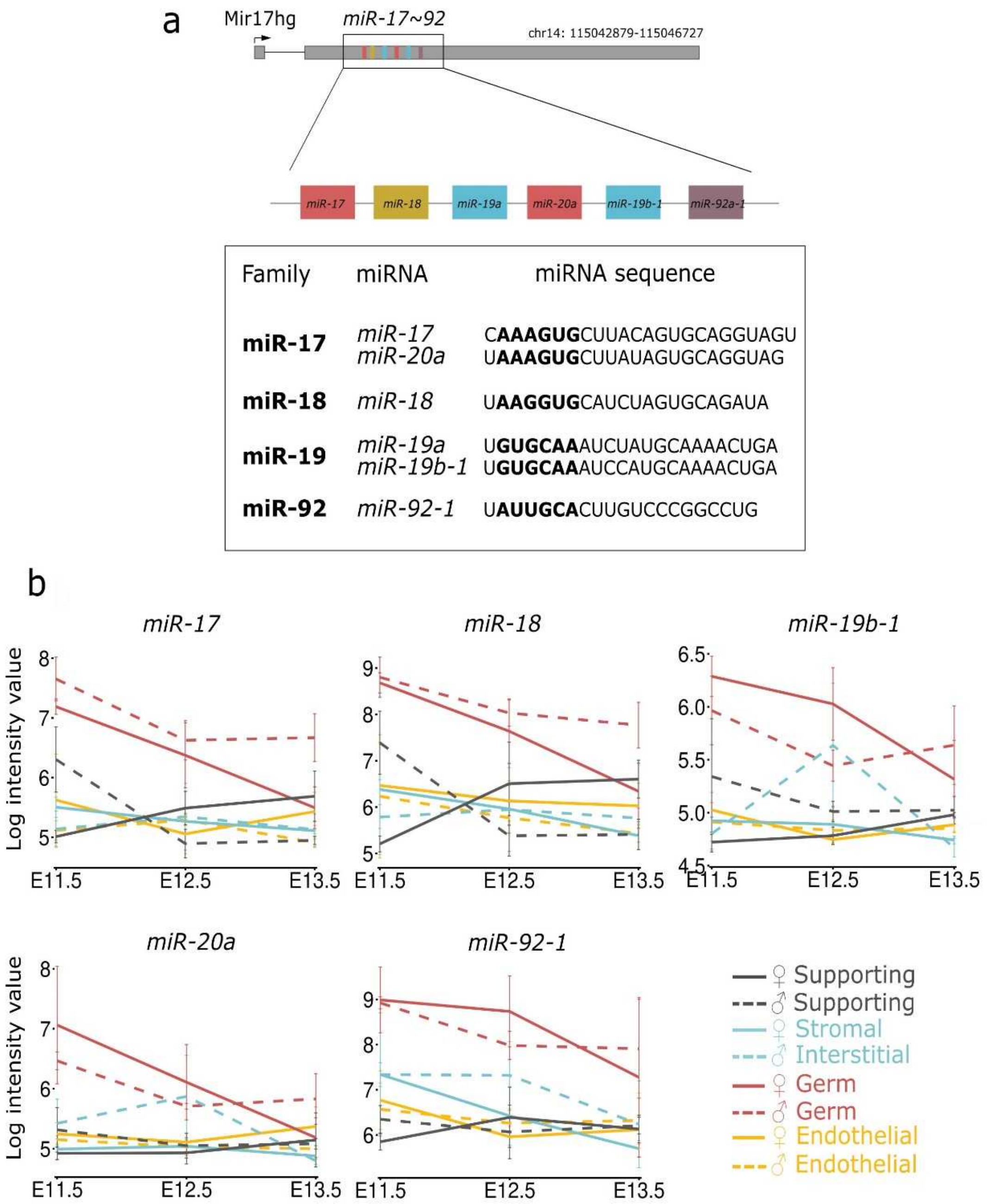

Supplementary Figure 1. **The mouse *miR-17~92* cluster. a**, Genomic organization of the mouse *miR-17~92* cluster (top). The members of the *miR-17~92* cluster are grouped in four families according to their seed sequences, the regions considered to be the most important for target selection (in bold; bottom). **b**, Expression of *miR-17~92* members in the main gonadal cell populations during mouse sex determination (E11.5) and early gonad differentiation. “Log intensity values", indicate the log-transformed, normalized intensity values from the microarray study by Jameson *et al.*, (2012) [Jameson, S. A. *et al.* Temporal transcriptional profiling of somatic and germ cells reveals biased lineage priming of sexual fate in the fetal mouse gonad. *PLoS Genet.* **8**, e1002575 (2012)].

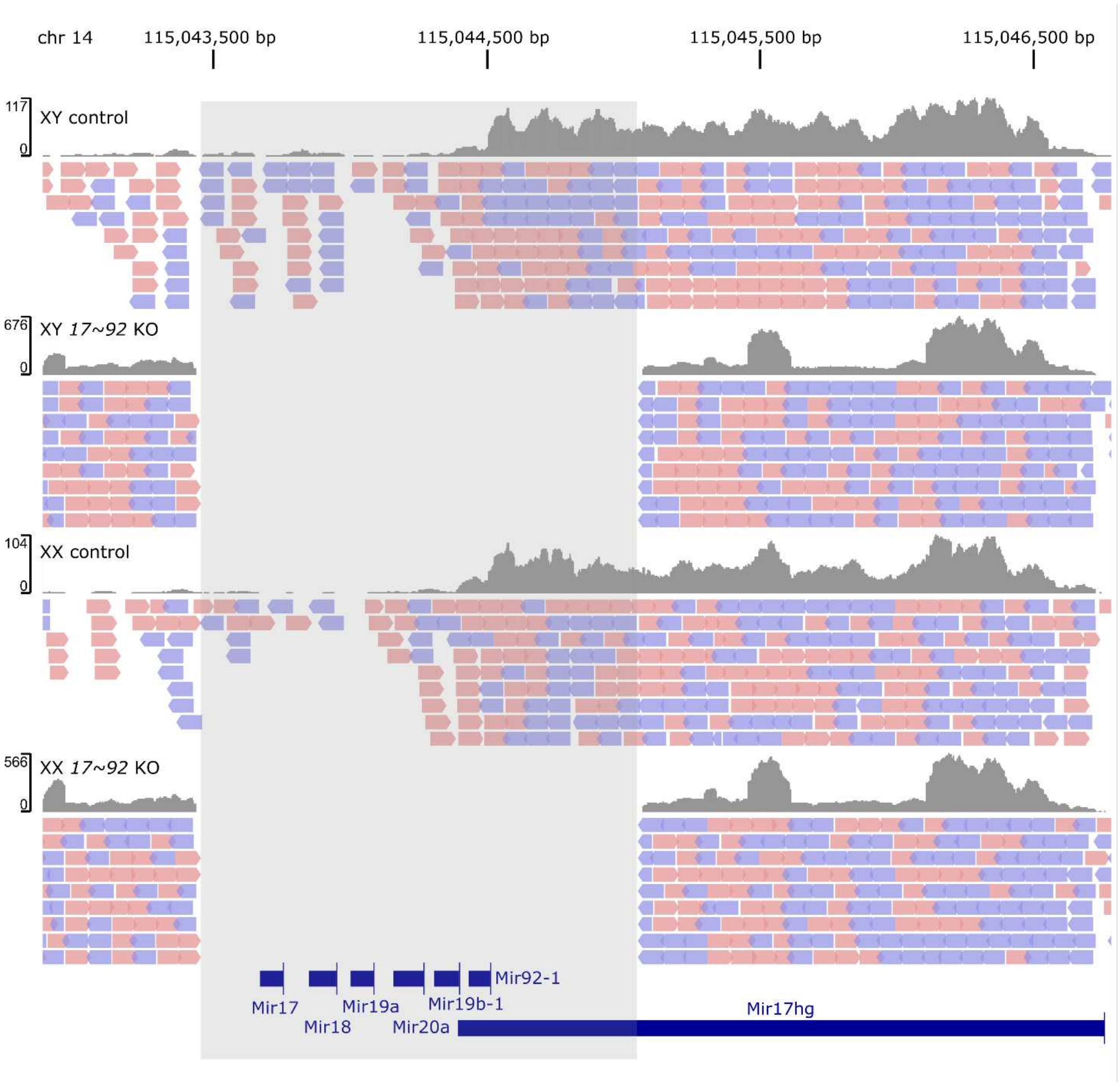

Supplementary Figure 2. Effective deletion of the *miR-17~92* cluster in mice. Integrative Genome Browser plot depicting RNA-seq reads from E11.5 gonads mapped to the *Mir17hg* locus. Genetic sex (XY or XX) and genotype (control or mutant) are depicted in the upper left of each track. Segments represent transcript reads mapped to the forward (red) or reverse (blue) DNA strand. The area marked in grey indicates the deleted region. Note that, unlike the control gonads, no transcript reads are found in the deleted region of the mutant gonads, demonstrating effective deletion. Mutant mice were generated using the Cre/LoxP system.

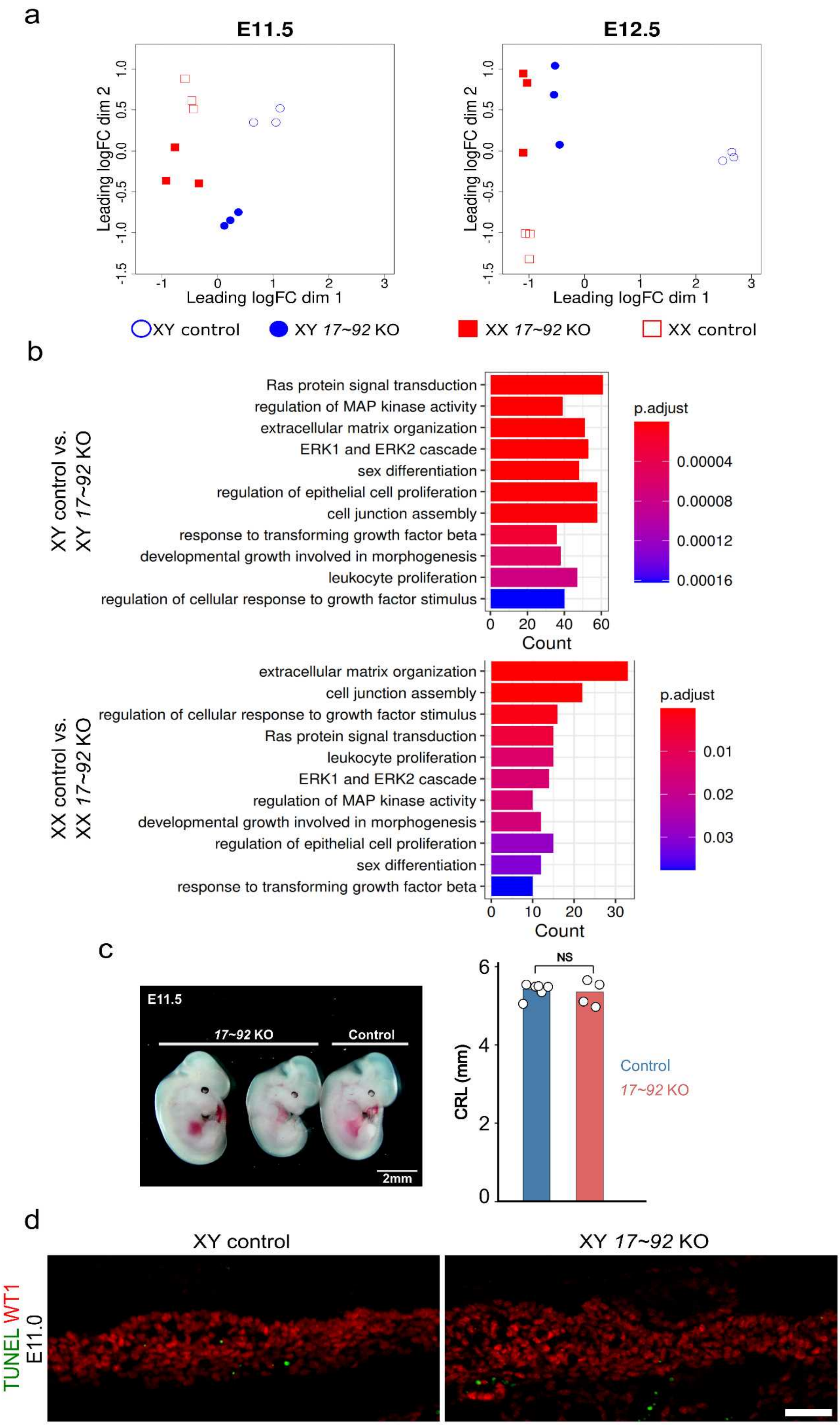

a
E11.5
E12.5
Leading logFC dim 2
Leading logFC dim 1
XY control
XY 17~92 KO
XX 17~92 KO
XX control
b
XY control vs. XY 17~92 KO
Ras protein signal transduction
regulation of MAP kinase activity
extracellular matrix organization
ERK1 and ERK2 cascade
sex differentiation
regulation of epithelial cell proliferation
cell junction assembly
response to transforming growth factor beta
developmental growth involved in morphogenesis
leukocyte proliferation
regulation of cellular response to growth factor stimulus
p.adjust
Count
XX control vs. XX 17~92 KO
extracellular matrix organization
cell junction assembly
regulation of cellular response to growth factor stimulus
Ras protein signal transduction
leukocyte proliferation
ERK1 and ERK2 cascade
regulation of MAP kinase activity
developmental growth involved in morphogenesis
regulation of epithelial cell proliferation
sex differentiation
response to transforming growth factor beta
p.adjust
Count
c
E11.5
17~92 KO
Control
2mm
NS
CRL (mm)
Control
17~92 KO
d
XY control
XY 17~92 KO
TUNEL WT1
E11.0

Supplementary Figure 3. Transcriptomic analyses and apoptotic cells detection in *miR-17~92* KO gonads. a, Multidimensional scaling plots of transcriptomic data from XX and XY control and *miR-17~92* KO gonads at E11.5 and E12.5. At E11.5 replicate samples of all conditions clustered close together, whereas at E12.5 the XY control group was clearly separated from the other samples. **b**, Gene Ontology analyses revealed common deregulated biological processes between controls and mutants in both XX and XY gonads. **c,** Body size comparison between control (n=6) and mutant (n=4) embryos at E11.5. No significant differences were observed in the crown-rump lengths (CRL). Two-tail t test, P=0.84457 **d,** TUNEL assay showed no difference in apoptosis between control and mutant gonads during early gonadal differentiation. Scale bar represents 50 µm. Mutant mice were generated using the Cre/LoxP system.

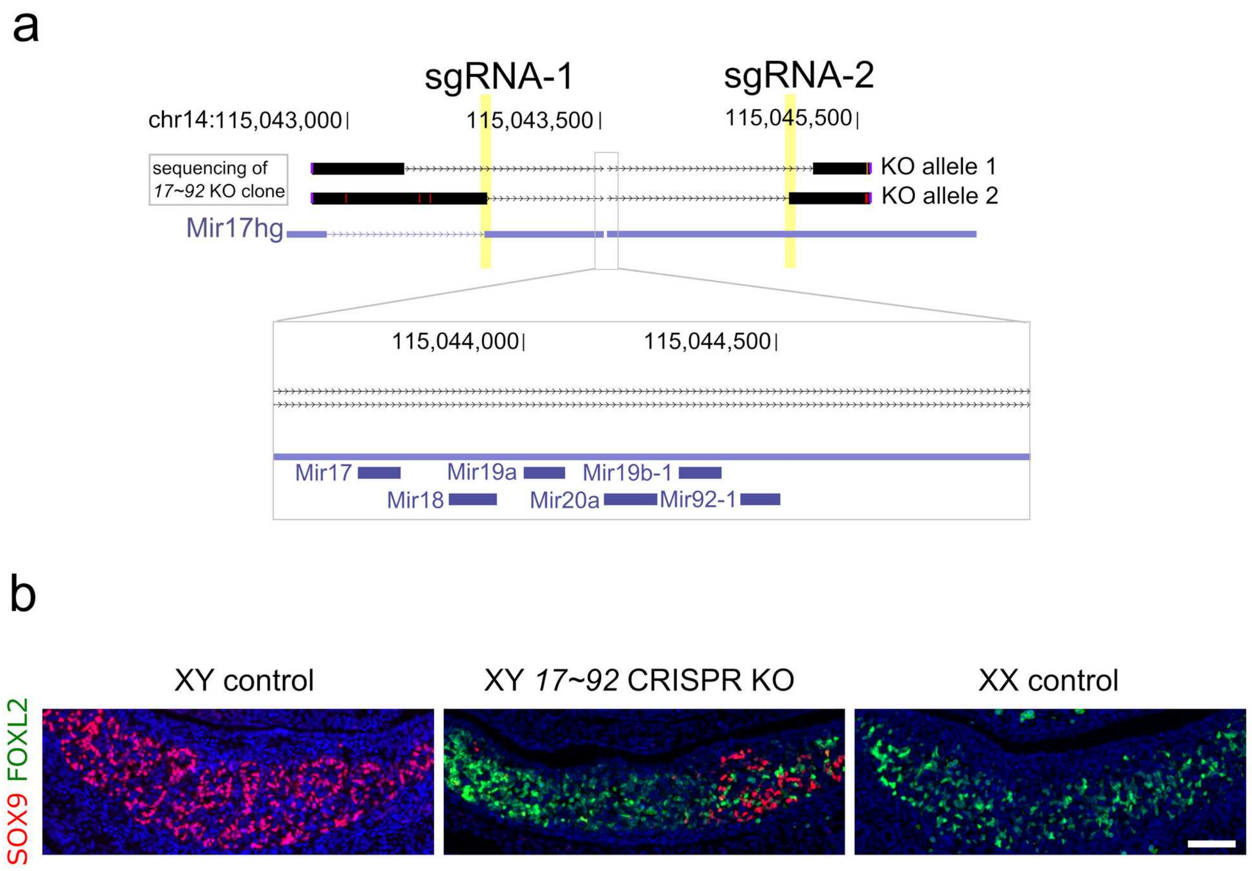

Supplementary Figure 4. Deletion of the *miR-17*~92 cluster by CRISPR/Cas. a, DNA sequence from *miR-17~92* CRISPR KO mESC clones (black) obtained by Sanger sequencing and mapped to the *Mir17hg* genome region (purple). Yellow lines show where the sgRNA guides were designed. Note that in both alleles the region containing the members of *miR-17~92* cluster is depleted. **b,** SOX9-FOXL2 double immunofluorescence in control and mutant gonads at E12. SOX9 is disappearing in XY *17~92* CRISPR KO gonad, while FOXL2 occupies most of the gonadal area. Scale bar represents 100 μm for all figures.

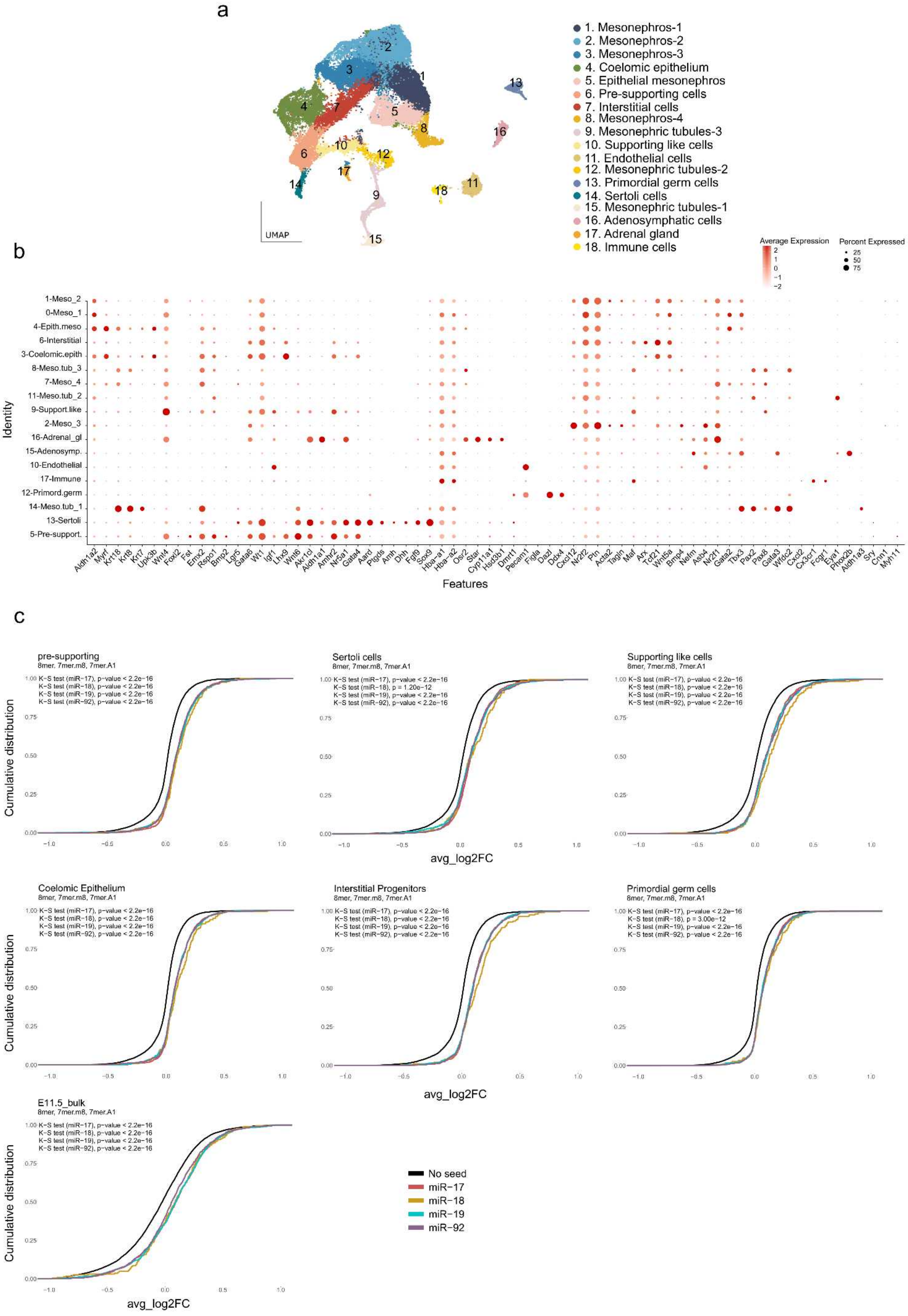
a
1. Mesonephros-1
2. Mesonephros-2
3. Mesonephros-3
4. Coelomic epithelium
5. Epithelial mesonephros
6. Pre-supporting cells
7. Interstitial cells
8. Mesonephros-4
9. Mesonephric tubules-3
10. Supporting like cells
11. Endothelial cells
12. Mesonephric tubules-2
13. Primordial germ cells
14. Sertoli cells
15. Mesonephric tubules-1
16. Adenosymphatic cells
17. Adrenal gland
18. Immune cells
UMAP
b
Average Expression
Percent Expressed
25
50
75
1-Meso_2
0-Meso_1
4-Epith.meso
6-Interstitial
3-Coelomic.epith
8-Meso.tub_3
7-Meso_4
11-Meso.tub_2
9-Support.like
2-Meso_3
16-Adrenal_gl
15-Adenosymp.
10-Endothelial
17-Immune
12-Primord.germ
14-Meso.tub_1
13-Sertoli
5-Pre-support.
Identity
Aldh1a2 Myrf Krt18 Krt8 Krt7 Upk3b Wnt4 Foxl2 Fst Emx2 Rspo1 Bmp2 Lgr5 Gata6 Wt1 Igf1 Lhx9 Wnt6 Akr1cl Aldh1a1 Amhr2 Nr5a1 Gata4 Aard Ptgds Amh Dhh Fgf9 Sox9 Hba-a1 Hba-a2 Osr2 Star Cyp11a1 Hsd3b1 Dmrt1 Pecam1 Figla Dazl Ddx4 Cxcl12 Nr2f2 Ptn Acta2 Tagln Maf Arx Tcf21 Wnt5a Bmp4 Nefm Asb4 Nr2f1 Gata2 Tbx3 Pax2 Pax8 Gata3 Wfdc2 Cxcl2 Cx3cr1 Fcgr1 Eya1 Phox2b Aldh1a3 Sry Cnn1 Myh11
Features
c
pre-supporting
8mer, 7mer.m8, 7mer.A1
K-S test (miR-17), p-value < 2.2e-16
K-S test (miR-18), p-value < 2.2e-16
K-S test (miR-19), p-value < 2.2e-16
K-S test (miR-92), p-value < 2.2e-16
Sertoli cells
8mer, 7mer.m8, 7mer.A1
K-S test (miR-17), p-value < 2.2e-16
K-S test (miR-18), p = 1.20e-12
K-S test (miR-19), p-value < 2.2e-16
K-S test (miR-92), p-value < 2.2e-16
Supporting like cells
8mer, 7mer.m8, 7mer.A1
K-S test (miR-17), p-value < 2.2e-16
K-S test (miR-18), p-value < 2.2e-16
K-S test (miR-19), p-value < 2.2e-16
K-S test (miR-92), p-value < 2.2e-16
Coelomic Epithelium
8mer, 7mer.m8, 7mer.A1
K-S test (miR-17), p-value < 2.2e-16
K-S test (miR-18), p-value < 2.2e-16
K-S test (miR-19), p-value < 2.2e-16
K-S test (miR-92), p-value < 2.2e-16
Interstitial Progenitors
8mer, 7mer.m8, 7mer.A1
K-S test (miR-17), p-value < 2.2e-16
K-S test (miR-18), p-value < 2.2e-16
K-S test (miR-19), p-value < 2.2e-16
K-S test (miR-92), p-value < 2.2e-16
Primordial germ cells
8mer, 7mer.m8, 7mer.A1
K-S test (miR-17), p-value < 2.2e-16
K-S test (miR-18), p = 3.00e-12
K-S test (miR-19), p-value < 2.2e-16
K-S test (miR-92), p-value < 2.2e-16
E11.5_bulk
8mer, 7mer.m8, 7mer.A1
K-S test (miR-17), p-value < 2.2e-16
K-S test (miR-18), p-value < 2.2e-16
K-S test (miR-19), p-value < 2.2e-16
K-S test (miR-92), p-value < 2.2e-16
Cumulative distribution
avg_log2FC
No seed
miR-17
miR-18
miR-19
miR-92

Supplementary Figure 5. scRNA-seq clustering, annotation and Cumulative Distribution Fraction (CDF) plots of gene expression. a, UMAP representation of single-cell data from XY control (E11.5), XY *miR-17-92* KO (E11.5 and E11.75) and XX (E11.5) control mouse gonads showing initial cluster identification and annotation. **b**, Expression of representative marker genes and cluster annotation. **c**, CDF plots of log2-fold changes of genes without (black) or with (coloured) 8mer, 7mer.m8, and 7mer.A1 matches (data obtained from https://www.targetscan.org) for members of the *miR-17~92* cluster in their 3'UTR using the bulk and scRNA-seq differential expression datasets at E11.5-E11.75. Mutant mice were generated using the CRISPR/Cas-tetraploid aggregation method, except for the E11.5_bulk CDF plots, where the Cre/loxP method was used.

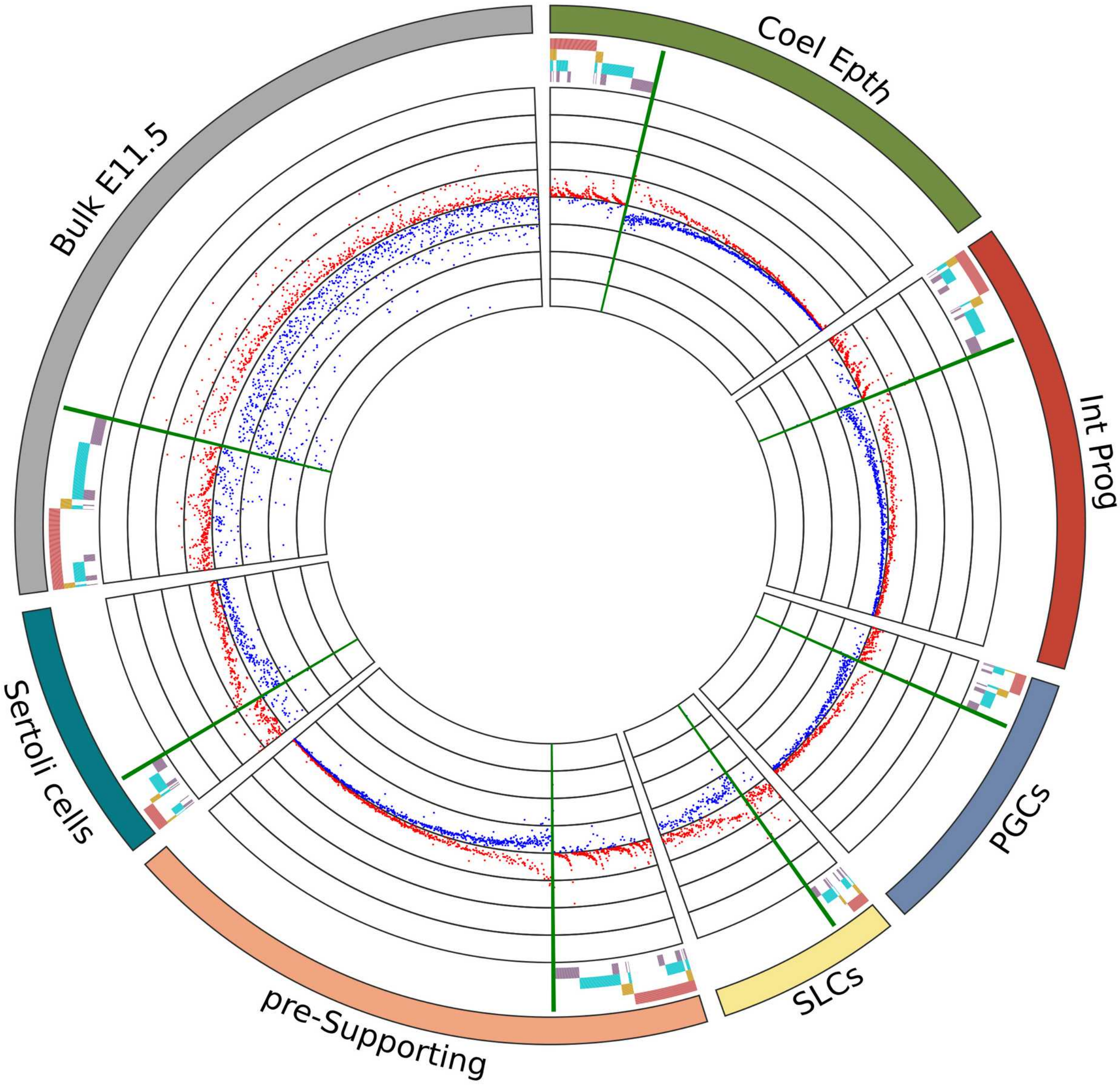

Supplementary Figure 6. Higher resolution image of the circos plot shown in Figure 4a. Coel. Epth., Coelomic Epithelium; Int. Prog., Interstitial Progenitors; PGCs, Primordial Germ Cells; SLCs, supporting-like cells. Mutant mice were generated using the CRISPR/Cas-tetraploid aggregation method.

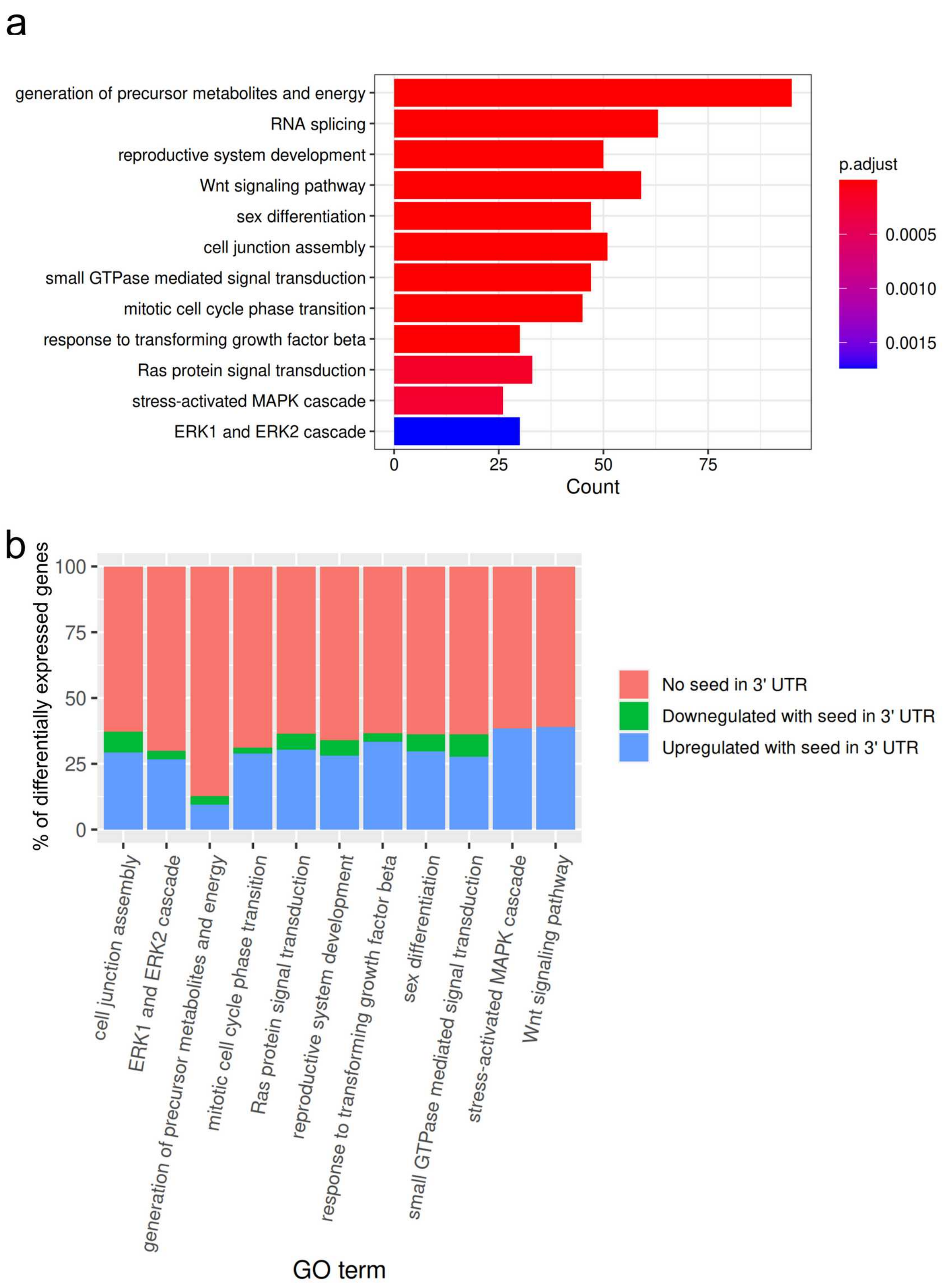

Supplementary Figure 7. Gene Ontology analysis of mutant pre-supporting cells. a, GO analysis using DEG between XY control and *miR-17~92* KO pre-supporting cells. **b,** Percentage of up- and downregulated *miR-17~92* target genes present in the GO categories shown in **a**. Mutant mice were generated using the CRISPR/Cas-tetraploid aggregation method.

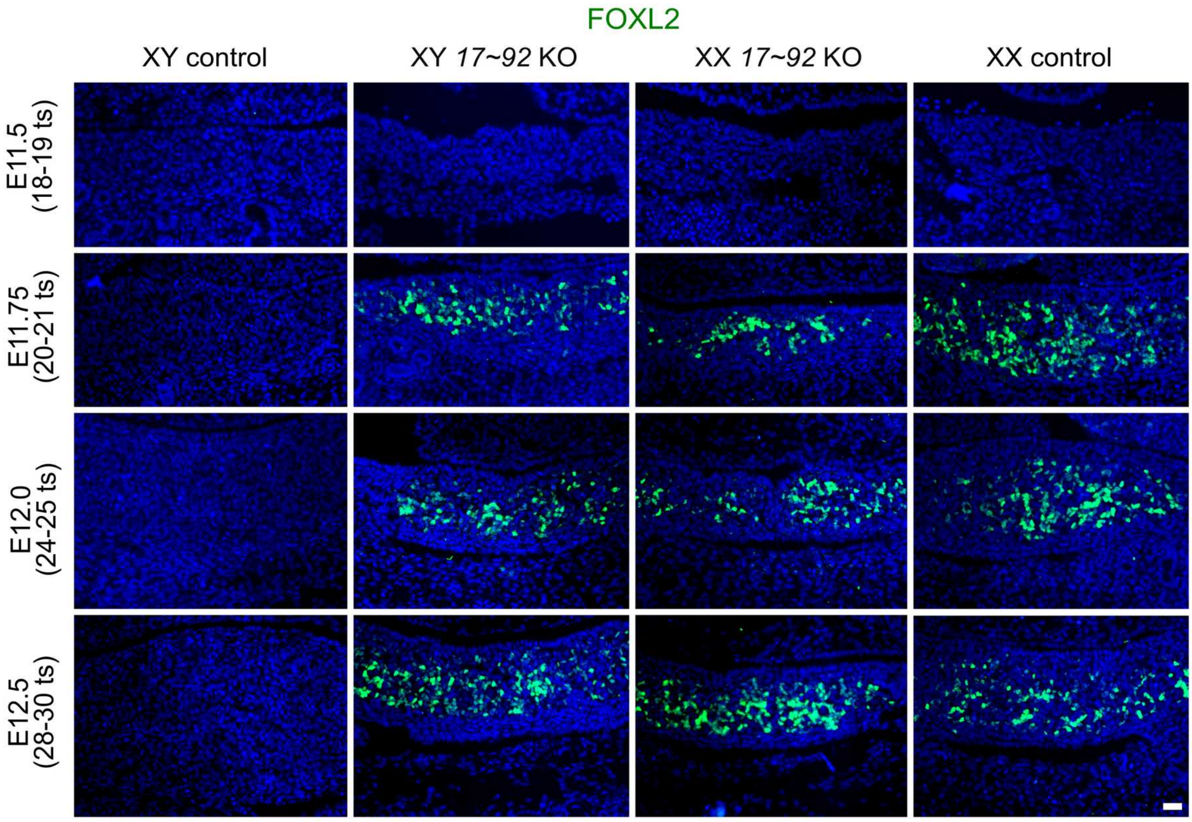

Supplementary Figure 8. FOXL2 expression in *miR-17~92* KO gonads. Immunofluorescence for FOXL2 in control and mutant gonads during (E11.5) and shortly after (E11.75-E12.5) the sex determination stage. Scale bar represents 50 µm. Mutant mice were generated using the Cre/LoxP system.

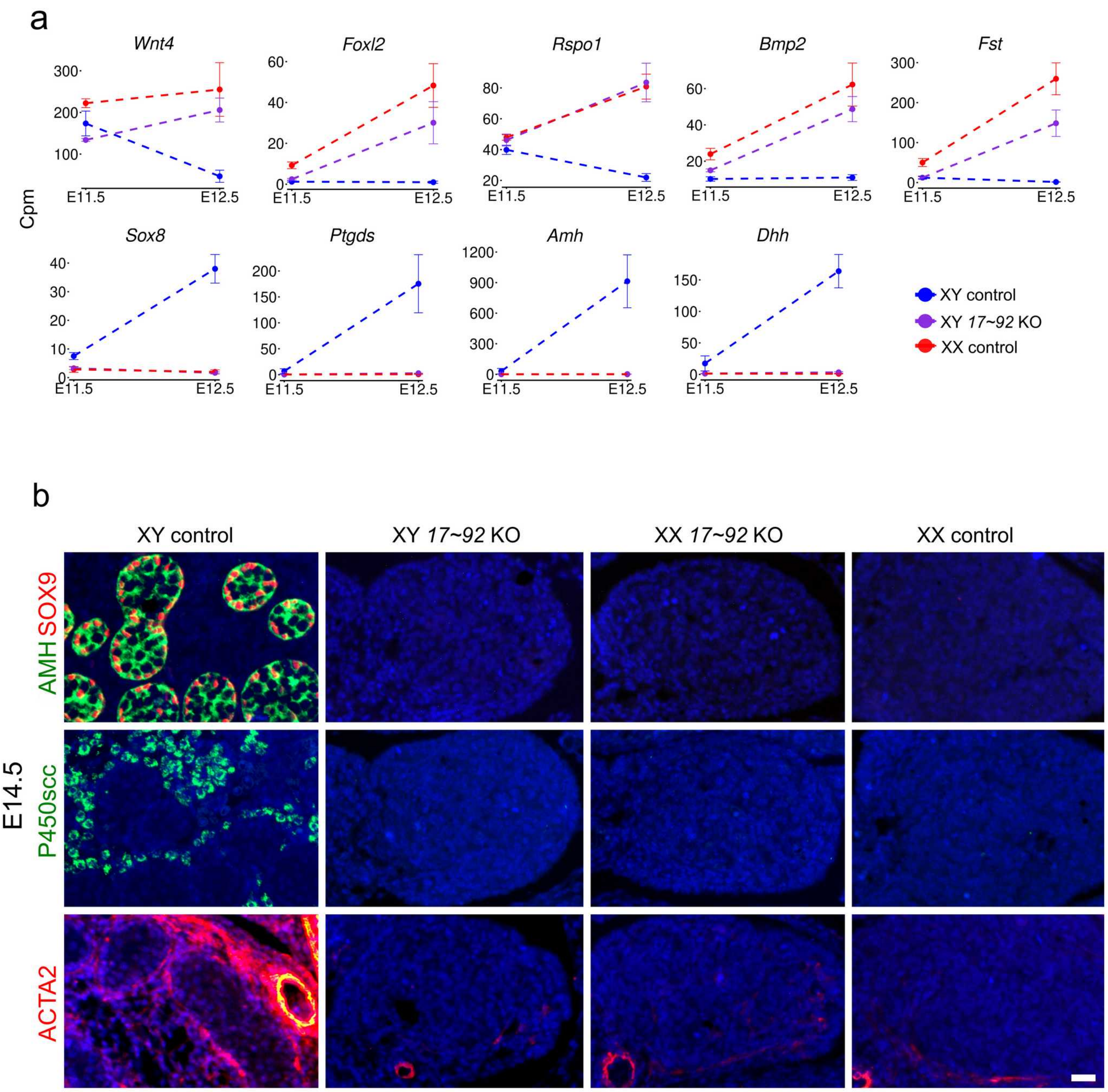

Supplementary Figure 9. Gonadal expression of marker genes after the sex determination stage. **a,** Gonadal Bulk RNA-seq transcripts levels (in Count per million, Cpm) of ovary-specific markers (*Wnt4*, *Foxl2*, *Rspo1*, *Bmp2*, *Fst*) and testis-specific markers (*Sox8*, *Ptgds*, *Amh*, *Dhh*), in control and mutant mice at E11.5 and E12.5. **b,** Immunofluorescence for testis-specific markers in control and mutant gonads at E14.5. At this stage, XY *miR-17~92* KO gonads show no expression for markers of Sertoli (SOX9 and AMH), Leydig (P450scc) and peritubular myoid cells (ACTA2). In addition, ACTA2 expression reveals the presence of the coelomic vessel in XY control gonads, but not in the other genotypes. Scale represents 20 µm. Mutant mice were generated using the Cre/LoxP system.

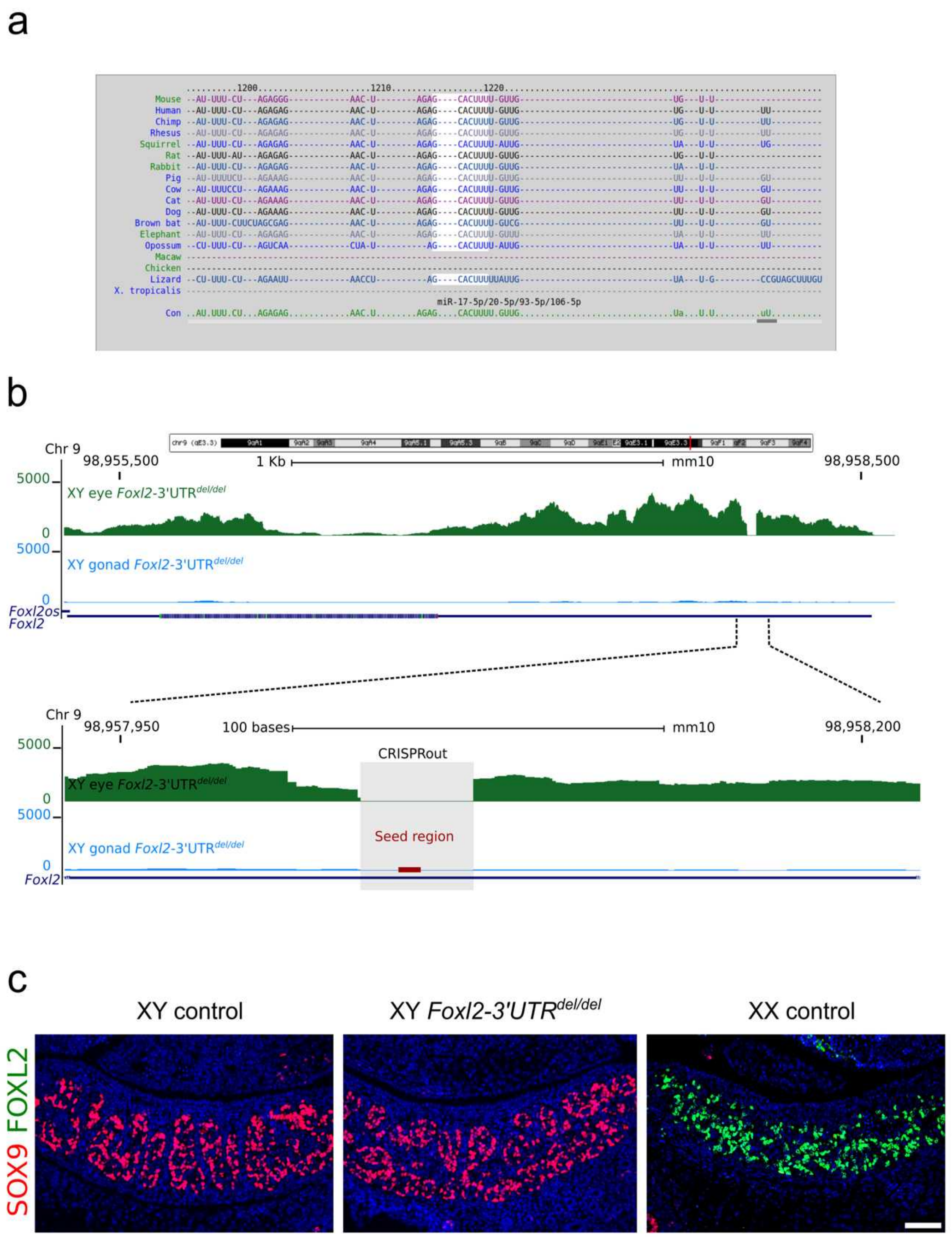

Supplementary Figure 10. Deletion of the *miR-17* seed family binding site in the 3' UTR of *Foxl2*. **a,** Predicted binding site for the *miR-17* seed family in the 3' UTR of *Foxl2* using TargetScan (http://www.targetscan.org/). **b,** Transcript reads mapped to the *Foxl2* genome region from embryonic eyes (top track) and gonad (bottom track) of XY mice with a deletion of the predicted *miR-17* seed family binding site in the 3' UTR of *Foxl2* (*Foxl2-3'-UTR*$^{del/del}$) at E12.5 (top). Note that the 3´UTR deletion does not affect *Foxl2* transcription in tissues where the gene is expressed, like the eye. High magnification indicating the deleted region (grey rectangle) and the seed position (red bar) in the 3' UTR (bottom). **c,** SOX9-FOXL2 double immunofluorescence in control and mutant gonads at E12.5. SOX9 is apparently normally expressed in XY *Foxl2-3'-UTR*$^{del/del}$ gonads, while FOXL2 is not present. Scale bar represents 100 μm for all figures.

a

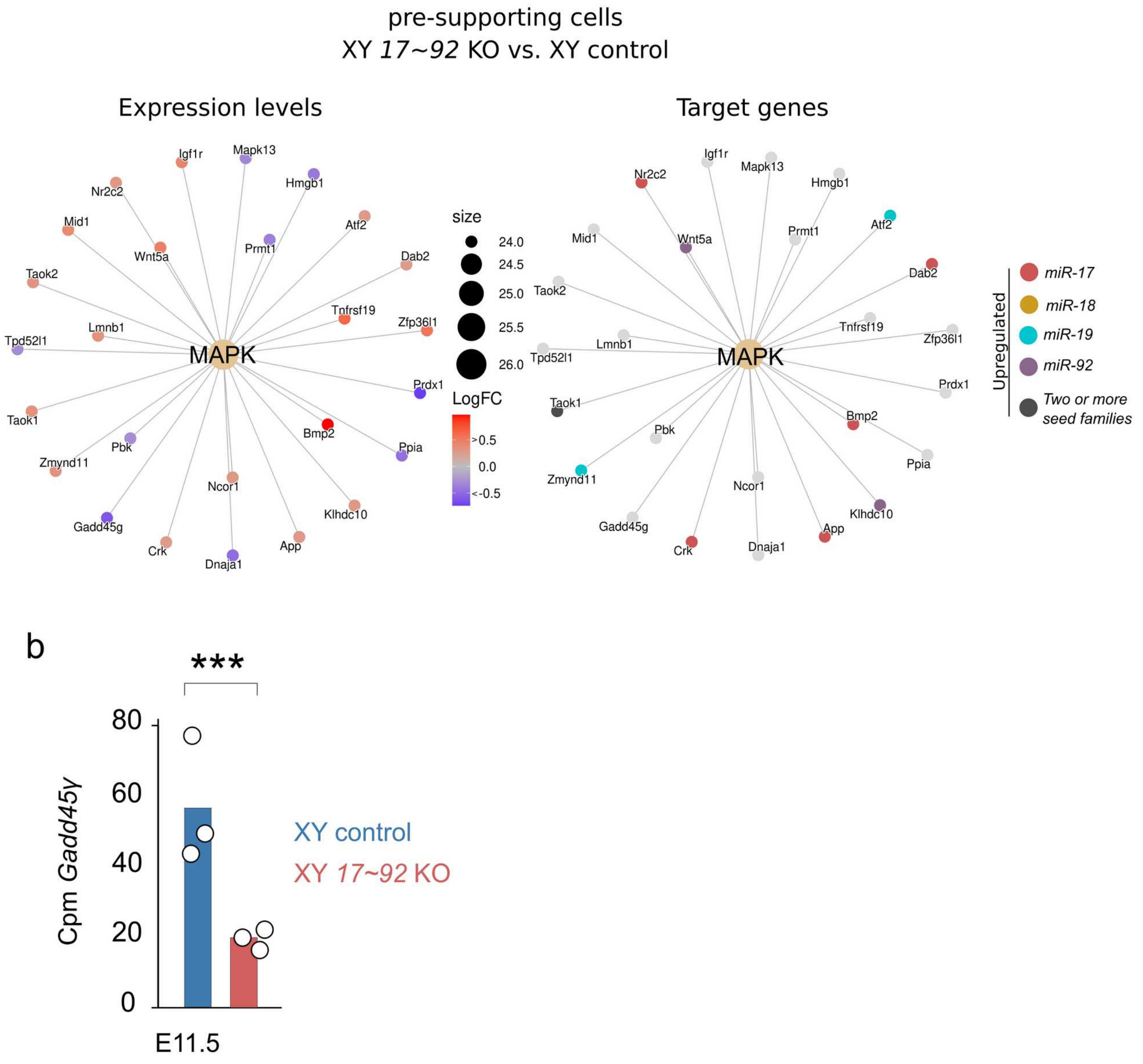

Supplementary Figure 11. MAPK signalling pathway in the pre-supporting cells. a, MAPK gene-concept network using DEGs between XY mutant and control pre-supporting cells at E11.5-E11.75. Log_2FCs (left) and predicted targets for the four *miR-17~92* seed families (right) are depicted. **b,** Gonadal expression levels of *Gadd45γ* in control and mutant testes at E11.5 (calculated from the Cpm data of the E11.5 bulk RNA-seq dataset; n=3 control and 3 mutant embryos). For statistical analysis we used the glmQLFit function of the bioconductor EdgeR package for multiple comparisons; FDR=0.0008755. Mutant mice were generated using both the CRISPR/Cas-tetraploid aggregation method (**a**) and the Cre-LoxP system (**b**).

Supplementary Note

Effects of the ablation of the *miR-17* binding site in the *Foxl2* 3'UTR

Previous studies have shown that overexpression of *Foxl2* during early testis development can cause male-to-female sex reversal[1 2]. Thus, differentiation of FOXL2^{+} pre-granulosa cells at early stages of gonadal development appears to be a key event underlying *miR-17~92*-associated sex reversal. Interestingly, *Foxl2* carries a functionally validated *miR-17* binding site in its 3´UTR[3] (Supplementary Fig. 10a) suggesting that early *Foxl2* upregulation in XY mutant gonads could be a direct consequence of *miR-17~92* ablation. To test this hypothesis, we deleted the *miR-17* binding site from the 3' UTR of *Foxl2* in XY mouse embryonic stem cells (mESC), and generated E11.5 and E12.5 embryos via tetraploid complementation assay (XY *Foxl2-3'-UTR*$^{del/del}$). The absence of the *miR-17* binding site did not affect *Foxl2* transcript stability in eye (Supplementary Fig. 10b). However, XY mutant embryos exhibited normal testis differentiation, with no evidence of *Foxl2* upregulation neither at the transcript nor at the protein level (Supplementary Fig. 10c; Supplementary Data Tables 20 and 21). Therefore, the upregulation of *Foxl2* at E12.0 in XY *miR-17~92* mutants is mostly an indirect effect derived from events occurring at earlier stages, likely associated to the low *Sox9* expression levels that are insufficient to antagonize the female pathway. In fact, *miR-17~92* ablation in Sertoli cells after the sex determination stage does not result in *Foxl2* upregulation[4]. Nevertheless, a transient down-regulation of *Foxl2* by *miR-17~92* in the testis shortly after the sex determination period cannot be completely excluded.

Supplementary References

1. Nicol, B. *et al.* Genome-wide identification of FOXL2 binding and characterization of FOXL2 feminizing action in the fetal gonads. *Hum. Mol. Genet.* **27**, 4273–4287 (2018).

2. Major, A. T., Ayers, K., Chue, J., Roeszler, K. & Smith, C. FOXL2 antagonises the male developmental pathway in embryonic chicken gonads. *J. Endocrinol.* JOE-19-0277.R1 (2019).

3. Rosario, R., Blenkiron, C. & Shelling, A. N. Comparative study of microRNA regulation on FOXL2 between adult-type and juvenile-type granulosa cell tumours in vitro. *Gynecol. Oncol.* **129**, 209–215 (2013).

4. Hurtado, A. *et al.* Sertoli cell-specific ablation of *miR-17-92* cluster significantly alters whole testis transcriptome without apparent phenotypic effects. *PLOS ONE* **13**, e0197685.
