# Supplementary material for: Complete male-to-female sex reversal in XY mice lacking the miR-17~92 cluster
Source: Nat Commun. 2024 May 7;15:3809. doi: 10.1038/s41467-024-47658-x (PMC11076593; doi:10.1038/s41467-024-47658-x)
Supplement: Supplementary file 3 — Description of Additional Supplementary Information [file 41467_2024_47658_MOESM3_ESM.pdf]

Description of Additional Supplementary Information

* Supplementary Data 1. Summary of the differential expression analysis.

* Supplementary Data 2. Differential expression analysis between XX and XY control gonads at E11.5

* Supplementary Data 3. Differential expression analysis between XY miR-17~92 KO gonads and XY control gonads at E11.5

* Supplementary Data 4. Differential expression analysis between XX miR-17~92 KO gonads and XX control gonads at E11.5

* Supplementary Data 5. Differential expression analysis between XX miR-17~92 KO gonads and XY miR-17~92 KO gonads at E11.5

* Supplementary Data 6. Differential expression analysis between XX and XY control gonads at E12.5

* Supplementary Data 7. Differential expression analysis between XY miR-17~92 KO gonads and XY control gonads at E12.5

* Supplementary Data 8. Differential expression analysis between XX miR-17~92 KO gonads and XX control gonads at E12.5

* Supplementary Data 9. Differential expression analysis between XX miR-17~92 KO gonads and XY miR-17~92 KO gonads at E12.5

* Supplementary Data 10. GO analysis on differential expressed genes between XY miR17~92 KO gonads and XY control gonads at E11.5

* Supplementary Data 11. GO analysis on differentially expressed genes between XX miR-17~92 KO gonads and XX control gonads at E11.5

* Supplementary Data 12. Differentially expressed genes between XY miR-17~92 KO and XY control gonads at E11.5 with 3' UTR predicted binding sites for the miR-17~92 seed families.

* Supplementary Data 13. Differentially expressed genes between E11.75 XY miR17~92 KO and E11.5 XY control Sertoli cells with 3' UTR predicted binding sites for the miR-17~92 seed families.

* Supplementary Data 14. Differentially expressed genes between E11.75 XY miR17~92 KO and E11.5 XY control preSupporting cells with 3' UTR predicted binding sites for the miR-17~92 seed families.

* Supplementary Data 15. Differentially expressed genes between E11.75 XY miR17~92 KO and E11.5 XY control Supporting like cells with 3' UTR predicted binding sites for the miR-17~92 seed families.

* Supplementary Data 16. Differentially expressed genes between E11.75 XY miR17~92 KO and E11.5 XY control Primordial germ cells with 3' UTR predicted binding sites for the miR-17~92 seed families.

* Supplementary Data 17. Differentially expressed genes between E11.75 XY miR17~92 KO and E11.5 XY control Interstitial cells with 3' UTR predicted binding sites for the miR-17~92 seed families.

* Supplementary Data 18. Differentially expressed genes between E11.75 XY miR17~92 KO and E11.5 XY control Coelomic epithelium cells with 3' UTR predicted binding sites for the miR-17~92 seed families.

* Supplementary Data 19. GO analysis on differentially expressed genes between E11.75 XY miR-17~92 KO and E11.5 XY control pre-supporting cells.

* Supplementary Data 20. Differential expression analysis between XY Foxl2-3'- UTR KO and XY control gonads at E11.5

* Supplementary Data 21. Differential expression analysis between XY Foxl2-3'- UTR KO and XY control gonads at E12.5

* Supplementary Data 22. Antibodies used in this study.
