# Supplementary material for: Complete male-to-female sex reversal in XY mice lacking the miR-17~92 cluster
Source: Nat Commun. 2024 May 7;15:3809. doi: 10.1038/s41467-024-47658-x (PMC11076593; doi:10.1038/s41467-024-47658-x)
Supplement: Supplementary file 6 — Reporting Summary [file 41467_2024_47658_MOESM6_ESM.pdf]

nature portfolio

Corresponding author(s): Rafael Jiménez, Francisco J. Barrionuevo and Darío G. Lupiáñez

Last updated by author(s): Mar 1, 2024

Reporting Summary

Nature Portfolio wishes to improve the reproducibility of the work that we publish. This form provides structure for consistency and transparency in reporting. For further information on Nature Portfolio policies, see our Editorial Policies and the Editorial Policy Checklist.

Statistics

For all statistical analyses, confirm that the following items are present in the figure legend, table legend, main text, or Methods section.

n/a	Confirmed	
☐	☒	The exact sample size (*n*) for each experimental group/condition, given as a discrete number and unit of measurement
☐	☒	A statement on whether measurements were taken from distinct samples or whether the same sample was measured repeatedly
☐	☒	The statistical test(s) used AND whether they are one- or two-sided<br>*Only common tests should be described solely by name; describe more complex techniques in the Methods section.*
☒	☐	A description of all covariates tested
☐	☒	A description of any assumptions or corrections, such as tests of normality and adjustment for multiple comparisons
☐	☒	A full description of the statistical parameters including central tendency (e.g. means) or other basic estimates (e.g. regression coefficient) AND variation (e.g. standard deviation) or associated estimates of uncertainty (e.g. confidence intervals)
☐	☒	For null hypothesis testing, the test statistic (e.g. *F*, *t*, *r*) with confidence intervals, effect sizes, degrees of freedom and *P* value noted<br>*Give P values as exact values whenever suitable.*
☒	☐	For Bayesian analysis, information on the choice of priors and Markov chain Monte Carlo settings
☒	☐	For hierarchical and complex designs, identification of the appropriate level for tests and full reporting of outcomes
☒	☐	Estimates of effect sizes (e.g. Cohen's *d*, Pearson's *r*), indicating how they were calculated

Our web collection on statistics for biologists contains articles on many of the points above.

Software and code

Policy information about availability of computer code

Data collection	No software was used for data collection
Data analysis	The bulk RNA-seq reads were mapped to the mm10 mouse genome and counted with the “align” and “featureCounts” function from the R subread package.<br>Analysis of differential gene expression was performed with edgeR.<br>Gene Ontology analysis was performed with the enrichGO function of the clusterProfiler bioconductor package.<br>For gene-concept analysis we used the cnetplot function of the same suite.<br>Prediction of target sites for the miR-17-92 seed families was performed using TargetScan (mouse version 8).<br>Differential expression was assessed with the limma R package.<br>Regarding single cell RNA-seq, each single cell library was aligned to the mm10 reference genome (mm10_v3.0.0, obtained from 10x Genomics) and transcriptome (modified to include a second exon of Sry and filtered with Cell Ranger Software; version 6.1.1) using default parameters.<br>The filtered counts matrices of all the samples with two technical replicates were merged using Seurat (version 4.0.2).<br>Data was scaled regressing the nCount_RNA (transcripts) and percentage of mitochondrial genes.<br>Clusters were obtained with a resolution of 1.2 (males) with the FindClusters function from Seurat.<br>Doublets were excluded using DoubletFinder (version 2.0.3).<br>In order to generate a single dataset with all samples, the Seurat objects were integrated using the Canonical Correlation analysis (CCA) method with 40 dimensions and using the 2000 most variable features.<br>Clusters were called at resolution 1 (females) with the FindClusters function from Seurat.<br>Markers for each cluster were identified using the FindMarkers function of Seurat with default parameters.

DEG lists between the clusters and samples of interest were generated using the Model-based Analysis of Single cell Transcriptomics (MAST) method implemented in Seurat.

For manuscripts utilizing custom algorithms or software that are central to the research but not yet described in published literature, software must be made available to editors and reviewers. We strongly encourage code deposition in a community repository (e.g. GitHub). See the Nature Portfolio guidelines for submitting code & software for further information.

Data

Policy information about availability of data

All manuscripts must include a data availability statement. This statement should provide the following information, where applicable:

- Accession codes, unique identifiers, or web links for publicly available datasets
- A description of any restrictions on data availability
- For clinical datasets or third party data, please ensure that the statement adheres to our policy

Transcriptomic data are publicly available on Gene Expression Omnibus under the following accession numbers: Serie: GSE225677; Subserie RNA-seq: GSE225675; Subserie scRNA-seq: GSE225676. A source data and two supplementary data files are provided with this paper. Correspondence and requests for materials should be addressed to: Darío G. Lupiáñez (dario.lupianez@csic.es); Rafael Jiménez (rjimenez@ugr.es); Francisco J. Barrionuevo (fjbarrio@go.ugr.es).

Research involving human participants, their data, or biological material

Policy information about studies with human participants or human data. See also policy information about sex, gender (identity/presentation), and sexual orientation and race, ethnicity and racism.

Reporting on sex and gender	No human material was used in this study
Reporting on race, ethnicity, or other socially relevant groupings	No human material was used in this study
Population characteristics	No human material was used in this study
Recruitment	No human material was used in this study
Ethics oversight	No human material was used in this study

Note that full information on the approval of the study protocol must also be provided in the manuscript.

Field-specific reporting

Please select the one below that is the best fit for your research. If you are not sure, read the appropriate sections before making your selection.

☒ Life sciences ☐ Behavioural & social sciences ☐ Ecological, evolutionary & environmental sciences

For a reference copy of the document with all sections, see nature.com/documents/nr-reporting-summary-flat.pdf

Life sciences study design

All studies must disclose on these points even when the disclosure is negative.

Sample size	In order to minimise the use of laboratory mice, for each experiment we used the minimum number of samples capable of providing statistically significant results (3 individuals per genotype). The software we used for the statistical analysis of the transcriptomic data was able to detect highly significant differences between genotypes (very low P-values) using samples of 3 individuals, demonstrating that this sample size is sufficient. These are acceptable sample sizes in this type of experiment (see: Khang, T. F., & Lau, C. Y. (2015). Getting the most out of RNA-seq data analysis. PeerJ, 3, e1360).
Data exclusions	In the case of bulk RNA-seq, only genes with 1 or more cpm (counts per million) in at least two of the samples were considered to be expressed and used for further analysis.<br>In the analysis of differential gene expression only genes with a false discovery rate (FDR) <0.05 were considered to be differentially expressed.<br>In the Gene Ontology analysis, general terms and redundant terms were not displayed.<br>Regarding single cell RNA-seq, the filtered counts matrices of all the samples with two technical replicates were merged using Seurat (version 4.0.2) before proceeding with downstream analyses.<br>For the male samples, once they were merged, thresholds for percentage of mitochondrial genes were set in order to filter low quality cells (Male control 11.5 = 10%, Male mutant 11.5 = 10%, Male mutant 11.75 = 9%). Also, blood cells were filtered from the analyses according to their percentage of beta globin transcripts (male control 11.5 = 1.43%, male mutant 11.5 = 0.39%, male mutant 11.75 = 0.73%). Finally, only cells with nCount_RNA < 30000 (transcripts) and nFeatures_RNA < 7000 (genes expressed) were considered.<br>For the female samples, the filtering was made by excluding those clusters with low quality (low nFeatures_RNA or high number of beta hemoglobin genes expressed, compared to the rest of the clusters). Doublets were excluded using DoubletFinder (version 2.0.3).

Replication	All experiments were replicated between 2 and 4 times in order to confirm reproducibility of our results. The number of replicates for each experiment has been indicated in the Methods section (several subsections).
Randomization	Samples (embryonic gonads) were grouped according to their genotype (wild type or mutant) and the genetic sex (XX or XY) of the animals from which they were obtained.
Blinding	To calculate the gonadal volume of several mouse embryos (4 control males, 2 control females, 3 mutant males and 4 mutant females at the 18-19 ts stage), two different researchers performed the necessary measurements on the computer using the same samples (gonad micrographs) independently. Because of the clear differences in size and shape between testes and ovaries at this developmental stage, the two researchers knew which type of gonad they were measuring, so complete blinding was not possible. On the other hand, since XX ovaries and XY mutant gonads (also ovaries due to sex reversal) were very similar, the two researchers could not clearly discriminate between them. For each sample, the mean of the two volume values obtained was used.

Reporting for specific materials, systems and methods

We require information from authors about some types of materials, experimental systems and methods used in many studies. Here, indicate whether each material, system or method listed is relevant to your study. If you are not sure if a list item applies to your research, read the appropriate section before selecting a response.

Materials & experimental systems

n/a	Involved in the study	
☐	☒	Antibodies
☒	☐	Eukaryotic cell lines
☒	☐	Palaeontology and archaeology
☐	☒	Animals and other organisms
☒	☐	Clinical data
☒	☐	Dual use research of concern
☒	☐	Plants

Methods

n/a	Involved in the study	
☒	☐	ChIP-seq
☒	☐	Flow cytometry
☒	☐	MRI-based neuroimaging

Antibodies

Antibodies used: Supplementary data 22 of the manuscript contains detailed information in this respect.

Validation:

ACTA2 Sigma A2547

https://www.sigmaaldrich.com/ES/en/product/sigma/a2547?utm_source=google&utm_medium=cpc&utm_campaign=20849242488&utm_content=157212547352&gclid=CjwKCAiAloavBhBOEiwAbtAJO720hNa0UtKTeicCWuWelG-nvXzEuk2s99fuDlmPT8r_N_8empH1ABoCstQQAvD_BwE

The Sigma website shows 8 citations in which the antibody has been used for ACTA2 immune-detection, some of them in mice. Six additional cases are shown here below in which the antibody has been used for ACTA2 immune-detection, some of them in mice, among many others, that can be found after a database search:

1) Arfian, N. et al. Endothelin converting enzyme-1 (ECE-1) deletion in association with Endothelin-1 downregulation ameliorates kidney fibrosis in mice. Life Sciences 258, 118223 (2020).
2) Barnes, E. A. et al. β1-Subunit of the calcium-sensitive potassium channel modulates the pulmonary vascular smooth muscle cell response to hypoxia. American Journal of Physiology-Lung Cellular and Molecular Physiology 315, L265–L275 (2018).
3) Barrionuevo, F. J. et al. Sox9 and Sox8 protect the adult testis from male-to-female genetic reprogramming and complete degeneration. eLife 5, e15635 (2016).
4) Di Lorenzo, M., Winge, S. B., Svingen, T., De Falco, M. & Boberg, J. Intrauterine exposure to diethylhexyl phthalate disrupts gap junctions in the fetal rat testis. Current Research in Toxicology 1, 5–11 (2020).
5) Epstein, J. A. et al. Migration of cardiac neural crest cells in Splotch embryos. Development 127, 1869–1878 (2000).

AMH, Santa Cruz Biotechnology, CA sc-6886 (https://www.scbt.com/p/mis-antibody-c-20)

The Santa Cruz Biotechnology website shows the references of more than 50 publications in which the antibody has been used of AMH immune-detection, most of them in mouse.

CYP11A1 (P450scc) Santa Cruz Biotechnology, CA sc-18043

This antibody has been widely used for CYP11A1 inmune-detection in mice, as shown in a database search. These are some examples:

1) Adedara, I. A., Nanjappa, M. K., Farombi, E. O. & Akingbemi, B. T. Aflatoxin B1 disrupts the androgen biosynthetic pathway in rat Leydig cells. Food and Chemical Toxicology 65, 252–259 (2014).
2) Ademi, H. et al. Deciphering the origins and fates of steroidogenic lineages in the mouse testis. Cell Reports 39, 110935 (2022).
3) Bergeron, F. et al. Phosphorylation of GATA4 serine 105 but not serine 261 is required for testosterone production in the male mouse. Andrology 7, 357–372 (2019).

4) Dadhich, R. K. et al. Identification of Live Germ-Cell Desquamation as a Major Mechanism of Seasonal Testis Regression in Mammals: A Study in the Iberian Mole (Talpa occidentalis)1. Biology of Reproduction 88, 101, 1–12 (2013).
5) Dai, T., Yang, L., Wei, S., Chu, Y. & Dan, X. The effect of gonadotropin-inhibitory hormone on steroidogenesis and spermatogenesis by acting through the hypothalamic–pituitary–testis axis in mice. Endocrine (2024) doi:10.1007/s12020-024-03690-x.

FOXL2, Abcam ab5096 (https://www.abcam.com/en-es/products/primary-antibodies/foxl2-antibody-ab5096#)

The Abcam's website shows the references of 47 publications in which the antibody has been used of FOXL2 immune-detection, most of them in mice.

Ki67 Abcam ab15580 (https://www.abcam.com/en-es/products/primary-antibodies/ki67-antibody-ab15580)

A total of 2642 citations can be found in a web search in which the antibody has been used of Ki67 immune-detection, many of them in mouse.

OCT4 Santa Cruz Biotechnology, CA sc-8628 (https://www.scbt.com/p/oct-3-4-antibody-n-19#citations)

The Santa Cruz Biotechnology website shows the references of 76 publications in which the antibody has been used of OCT4 immune-detection, most of them in mice.

can be found 76 citations in which the antibody has been used of OCT4 immune-detection, many of them in mice.

SOX9 MERCK Millipore AB5535 (https://www.emdmillipore.com/US/en/product/Anti-Sox9-Antibody,MM_NF-AB5535?bd=1#)

In the Application Notes section of this website, more than 10 citations can be found in which the antibody has been used of SOX9 immune-detection, many of them in mouse.

SRY, kindly provided by Dr. Dagmar Wilhelm (University of Melbourne)

This antibody was initially described in : Wilhelm, D. et al. Sertoli cell differentiation is induced both cell-autonomously and through prostaglandin signaling during mammalian sex determination. Developmental Biology 287, 111–124 (2005).

Since then, it has been widely used for mouse SRY immunedetection. These are some examples:

1) Bagheri-Fam, S. et al. Testis Determination Requires a Specific FGFR2 Isoform to Repress FOXL2. Endocrinology 158, 3832–3843 (2017).
2) Bradford, S. T., Wilhelm, D. & Koopman, P. Comparative Analysis of Anti-Mouse SRY Antibodies. Sexual Development 1, 305–310 (2008).
3) Bradford, S. T. et al. A cell-autonomous role for WT1 in regulating Sry in vivo. Human Molecular Genetics 18, 3429–3438 (2009).
4) Callier, P. et al. Loss of Function Mutation in the Palmitoyl-Transferase HHAT Leads to Syndromic 46,XY Disorder of Sex Development by Impeding Hedgehog Protein Palmitoylation and Signaling. PLOS Genetics 10, e1004340 (2014).
5) Hiramatsu, R. et al. A critical time window of Sry action in gonadal sex determination in mice. Development 136, 129–138 (2009).

SYCP3, Serum K921, provided by Dr. José Luis Barbero (Centro de Investigaciones Biológicas Margarita Salas / CSIC, Madrid)

This antibody was initially described in Prieto, I. et al. Cohesin component dynamics during meiotic prophase I in mammalian oocytes. Chromosome Res 12, 197–213 (2004).

Since then, it has been widely used for mouse SYCP3 immunedetection. Here are some examples:

1) Felipe-Medina, N. et al. A missense in HSF2BP causing primary ovarian insufficiency affects meiotic recombination by its novel interactor C19ORF57/BRME1. eLife 9, e56996 (2020).
2) Felipe-Medina, N. et al. Ubiquitin-specific protease 26 (USP26) is not essential for mouse gametogenesis and fertility. Chromosoma 128, 237–247 (2019).
3) Felipe-Medina, N. et al. Ubiquitin-specific protease 26 (USP26) is not essential for mouse gametogenesis and fertility. Chromosoma 128, 237–247 (2019).
4) Franca, M. M. et al. A truncating variant of RAD51B associated with primary ovarian insufficiency provides insights into its meiotic and somatic functions. Cell Death Differ 29, 2347–2361 (2022).
5) Krasikova, A., Barbero, J. L. & Gaginskaya, E. Cohesion proteins are present in centromere protein bodies associated with avian lampbrush chromosomes. Chromosome Res 13, 675–685 (2005).

WT1, DAKO M3561 (clone 6F-H2) (https://www.citeab.com/antibodies/2414754-m3561-wilms-tumor-1-wt1-protein)

This DAKO website shows 101 citations in which the antibody has been used of WT1 immune-detection, many of them in mouse.

Animals and other research organisms

Policy information about studies involving animals; ARRIVE guidelines recommended for reporting animal research, and Sex and Gender in Research

Laboratory animals	Mirc1tm1.1Tyj/J<br><br>These mice have a C57BL/6 × 129S4/SvJae background and were acquired at the Jackson Laboratory (Bar Harbor, ME, USA; Stock 008458). Ref: Ventura, A. et al. Targeted Deletion Reveals Essential and Overlapping Functions of the miR-17?92 Family of miRNA Clusters. Cell 132, 875–886 (2008). Age: 2.5-3 months.<br><br>Tg(CAG-cre)1Nagy mice (MGI:3586452)<br><br>These mice have a NMRI background and were obtained from the CNB-CSIC, Centro Nacional de Biotecnologia, Madrid, Spain (EMMA Id EM:04371). Ref: Belteki, G. et al. Conditional and inducible transgene expression in mice through the combinatorial use of Cre-mediated recombination and tetracycline induction. Nucleic Acids Res. 33, e51–e51 (2005). Age: 2.5-3 months.<br><br>Mice carrying the homozygous deletion for miR-17~92 and mice bearing a deletion of the miR-17 seed sequence within the 3' UTR of Foxl2 using CRISPR/Cas9 technology:<br><br>For tetraploid aggregation, stud and vasectomised males and donor and foster females were CD1. (age > 2 months). The mutant embryos were C57BL/6J-129. These strains are usually available in the animal facility of the Max-Delbrück Center for Molecular Medicine, Berlin, Germany.<br><br>Mice were housed under Specific Pathogen-Free (SPF) conditions in the animal facilities of the Center for Biomedical Research (University of Granada, Granada, Spain) and the Max-Delbrück Center for Molecular Medicine, Berlin, Germany. The animals had ad libitum access to food and water and were kept in groups. The occupancy density of the cages (micro-ventilated) was based on legal requirements. The cages were kept at a temperature of 22 +/- 2°C, a humidity of 55 +/- 10% and a 12-hour/12 hour darck light cycle. The animals were provided with activity elements in the form of nest building material and hiding places.
Wild animals	No wild animals were used in this study
Reporting on sex	We analysed both XX and XY individuals of the two genotypes (wild type and mutant) and their sexual phenotype was studied in order to detect cases of sex reversal.
Field-collected samples	No samples collected in the field were used in this study
Ethics oversight	All animal experiments in this study were approved by the University of Granada Ethics Committee for Animal Experimentation and the regional government, "Junta de Andalucía" (exp. No: 16/12/2021/188) and by the Landesamt für Gesundheit und Soziales, Berlin, Germany under license number G0111/17, and were performed in accordance with the relevant guidelines and regulations dictated by these Committees.

Note that full information on the approval of the study protocol must also be provided in the manuscript.

Plants

Seed stocks	No plan material was used in this study
Novel plant genotypes	No plan material was used in this study
Authentication	No plan material was used in this study
